# When Differences Ignite Speaking Up: Contrasting Effects of Attitude Dissimilarity and Perceived Status Conflict on Employee Voice

**DOI:** 10.3390/bs15121714

**Published:** 2025-12-11

**Authors:** Yumi Ko, Myung-Ho Chung, Jeeyoung Kim

**Affiliations:** Ewha School of Business, Ewha Womans University, 52-Ewhayeodae-gil, Seodaemun-gu, Seoul 03760, Republic of Korea; myhoc@ewha.ac.kr (M.-H.C.); jeeyoungkim@ewha.ac.kr (J.K.)

**Keywords:** attitudinal dissimilarity, social comparison, attribution theory, status conflict, voice behavior

## Abstract

Drawing on social comparison and attribution theories, this study examines how employees’ attitudinal dissimilarity within work groups shapes their willingness to speak up or remain silent. We conceptualize dissimilarity in psychological ownership and job stress as individual-level differences that trigger internal attributions, leading employees to direct attention inward and reduce their likelihood of speaking up. In contrast, dissimilarity in perceived status conflict, an individual-level perception of a structural feature of the group, induces external attributions toward the social system, motivating employees to express voice aimed at preserving or challenging the status quo. Using multi-source data from 202 employees nested in 39 work groups in South Korea, hierarchical regression analyses support all proposed hypotheses: individual-level dissimilarities are negatively related and structural-level dissimilarity is positively related to voice. These findings reveal that the behavioral consequences of difference depend on where attribution is directed—toward the self or the system. The study contributes to the voice literature by integrating attributional reasoning into social comparison processes and by identifying two forms of attitudinal minorities: invisible minorities who remain silent, and boisterous minorities who speak up for change.

## 1. Introduction

Employee voice—the discretionary communication of suggestions, concerns, or ideas intended to improve the organization—has long been recognized as a critical driver of innovation, learning, and adaptation. Speaking up enables organizations to detect errors early, enhance decision quality, and sustain continuous improvement (Detert & Burris, 2007; Morrison & Milliken, 2000). Yet from an employee’s perspective, voicing is inherently risky. Raising issues or challenging established practices can invite negative evaluations from supervisors or peers, potentially threatening one’s social standing or relationships within the group (Morrison, 2011, 2023). Consequently, many employees with valuable insights choose to remain silent, creating what scholars have termed a “spiral of silence.” This paradox—voice as both valuable and vulnerable—underscores the need to better understand the conditions under which employees decide to speak up or stay quiet (Joseph & Shetty, 2022; Dehkharghani et al., 2023).

Despite extensive research on employee voice, most studies have concentrated on its motivational antecedents—such as psychological safety, leadership, or proactivity—while paying far less attention to how employees’ relative standing within their team shapes their willingness to speak up. Existing diversity and voice research has largely emphasized surface-level differences (e.g., gender, age, tenure) or absolute individual traits rather than relational dissimilarities in underlying attitudes. Yet, in daily workgroups, employees constantly evaluate how their values, emotions, and cognitions compare with others. These subtle discrepancies in psychological ownership, job stress, or perceptions of status conflict may quietly but powerfully determine who feels safe to express ideas and who withholds them, shaping employees’ overall willingness to express concerns or suggestions. Moreover, prior studies have seldom integrated social comparison theory and attribution theory to provide a theoretical lens for understanding how employees interpret such dynamics. Addressing these gaps, this study explores how attitudinal dissimilarity— both as an individual-level difference in personal work attitudes and an individual-level difference in perceptions of a structural feature of the group (status hierarchy)—influences employee voice. In doing so, it advances understanding of voice as a socially embedded, attribution-driven behavior, rather than a purely dispositional or contextual act.

Building on this perspective, we develop a theoretical model that explains when and why attitudinal dissimilarity leads employees either to stay silent or to speak up. Drawing on social comparison theory (Festinger, 1954) and attribution theory (Weiner et al., 1972), we argue that the psychological meaning employees attach to their differences within a group determines the direction of their behavioral response. Building on social comparison theory, we view work groups as important comparison contexts in which employees continually evaluate the similarity and difference in their attitudes relative to salient others (Festinger, 1954). Social comparison theory suggests that such evaluations are not neutral; they shape how individuals define their standing in the group and regulate their subsequent behaviors. Attribution theory further explains how employees interpret the causes of these similarities and differences. According to attribution theory (Weiner et al., 1972), individuals can locate the cause of a discrepancy either internally (e.g., “something about me”) or externally (e.g., “something about the situation or system”), and these causal inferences guide emotional reactions and behavioral responses. Specifically, dissimilarities in individual attitudes—such as psychological ownership and job stress—are likely to evoke internal attributions that focus attention on the self (“I am different because of me”), thereby inhibiting voice. In contrast, dissimilarities in structural perceptions, such as perceived status conflict, tend to trigger external attributions directed toward the social environment (“This difference reflects the group’s hierarchy”), which motivate employees to express themselves in an effort to preserve or challenge the status quo. By distinguishing these two attributional pathways, our model highlights how different forms of dissimilarity can lead to contrasting behavioral outcomes in terms of employees’ overall likelihood of speaking up, within the same social comparison process.

## 2. Theory and Hypotheses

### 2.1. Attitudinal Dissimilarity and Employee Voice

Research on diversity and voice has long recognized that differences among group members shape interpersonal interactions and behavioral outcomes. Early studies primarily focused on surface-level diversity—such as gender, age, tenure, and ethnicity—which are visible and easily categorized (Williams & O’Reilly, 1998). However, more recent work highlights that deep-level diversity, reflected in members’ attitudes, values, and cognitions, exerts a stronger and more enduring influence on group functioning (Harrison et al., 1998; Triana et al., 2021a). While demographic diversity and attitudinal diversity are conceptually related, they do not necessarily tap the same underlying processes. Demographic attributes such as gender, age, or ethnicity are often proxies for broader socialization experiences that may shape values and work attitudes over time. However, deep-level attitudes represent more proximal psychological states that directly regulate how individuals interpret their work environment and their relative standing in the team. Recent work suggests that demographic and deep-level diversity can generate distinct interactional mechanisms and outcomes (e.g., Harrison & Klein, 2007; van Dijk et al., 2012). We also argue that the two lines of research—demographic dissimilarity and attitudinal dissimilarity—capture related but not identical latent traits. Demographic attributes primarily reflect structural and externally visible categories, whereas attitudinal characteristics capture internalized, psychological orientations toward work that develop through personal experiences, sensemaking, and micro-level interactions. In this sense, attitudinal dissimilarity is not merely a proxy for demographic differences but represents a distinct form of heterogeneity that can uniquely influence interpersonal perceptions and group dynamics. Specifically, by focusing on attitudinal dissimilarity in psychological ownership, job stress, and perceived status conflict, our study isolates the effects of these more immediate, psychologically grounded differences above and beyond demographic categories, thereby offering a more nuanced account of how “being different” is experienced in everyday voice decisions. Deep-level dissimilarities shape how individuals interpret their relationships, evaluate themselves, and decide whether to share or withhold their opinions. In essence, similarity among members facilitates communication and trust, whereas dissimilarity can create subtle barriers that inhibit openness and information exchange (Muchinsky & Monahan, 1987). These dynamics are particularly relevant to voice behavior, which is inherently social and evaluative: when expressing one’s opinion, employees must anticipate how others will interpret or react to it.

Drawing from social comparison theory (Festinger, 1954), we argue that individuals continuously evaluate how their attitudes and experiences align with or deviate from those of their coworkers. In this sense, we define “coworkers” as members of the same work group or team with whom employees share daily interactions and task interdependence. Such comparisons shape one’s perceived position within the team—whether as part of the majority or a deviant minority—and influence subsequent emotional and behavioral responses. When employees recognize that their attitudes differ from those of their peers, they engage in attributional reasoning to understand why this difference exists. Following attribution theory (Weiner et al., 1972), individuals may interpret the cause of dissimilarity as stemming from internal factors (e.g., their own ability, values, or effort) or external factors (e.g., features of the group or hierarchy). These attributions, in turn, guide whether employees turn their attention inward—making them more cautious and less likely to voice—or outward—motivating expressions that challenge or reaffirm the status quo. Accordingly, attitudinal dissimilarity can function as a social signal that is associated with distinct patterns of attributional reasoning and behavioral outcomes in the context of voice.

Although employees’ subjective perceptions of being similar or different are important, we, in this study, focus on quasi-objective dissimilarity because it captures the structural position of an individual within the attitudinal distribution of the team. Objective deviation from the team mean reflects the extent to which an employee occupies an attitudinal minority position, which is a prerequisite for subjective experiences of being different and for social comparison processes to unfold. Thus, our measure of dissimilarity provides a structural basis for the internal or external attributions that employees subsequently make about their difference.

Moreover, we focus on attitudinal (dis)similarity within the work group. By coworkers, we specifically refer to members of the same team who share common tasks and interact on a regular basis. Social comparison processes are most salient among proximal, task-interdependent others, making the team the theoretically appropriate reference group for assessing relational differences in work-related attitudes. Accordingly, all dissimilarity scores are calculated relative to other members within the same work group rather than to employees in the broader organization.

### 2.2. Invisible Minorities in Work-Related Attitudes

#### 2.2.1. Dissimilarity in Psychological Ownership and Employee Voice

Psychological ownership represents the feeling that one’s work or organization “belongs” to them—a sense of personal identification and responsibility toward the target (Pierce et al., 2003). When an employee’s level of psychological ownership diverges from that of their peers, this difference is likely to be internally attributed, as individuals interpret such divergence as reflecting their own effort, involvement, or commitment. According to attribution theory, internal attributions direct attention inward, evoking self-focused emotions such as guilt, pride, or self-doubt (Weiner et al., 1972). These emotions tend to heighten self-awareness and risk aversion, which in turn discourage individuals from expressing views that might expose them as different. From a social comparison perspective, these employees may prefer to remain “under the radar,” minimizing attention to their unique stance in order to maintain relational harmony within the group. Bowen and Blackmon (2003) also noted that individuals may hesitate to disclose their true identities and perspectives for fear of impacting their social exchanges within the organization. When people hide their views, they not only withhold ideas and opinions but also conform to others by suppressing their own viewpoints to avoid displeasing others or facing social exclusion, thereby reducing their voice behavior.

**H1.**  *An employee’s dissimilarity in psychological ownership will be negatively related to their voice behavior.*

#### 2.2.2. Dissimilarity in Job Stress and Employee Voice

Job stress reflects an individual’s psychological response to perceived work demands and constraints (Jamal, 2007). Employees who experience stress levels that differ substantially from those of their peers may interpret this difference as stemming from personal limitations—such as inadequate coping ability or inefficiency. Consistent with internal attribution processes, such employees are likely to focus attention on the self rather than the situation, leading to feelings of vulnerability and withdrawal. From a social comparison standpoint, perceiving oneself as less resilient or less balanced than others can amplify the fear of negative evaluation. Consequently, rather than voicing concerns that might reveal their distinct experience, these employees often refrain from speaking up as a self-protective strategy.

**H2.**  *An employee’s dissimilarity in job stress will be negatively related to their voice behavior.*

### 2.3. Boisterous Minorities in Perceived Status Conflict

#### Dissimilarity in Perceived Status Conflict and Employee Voice

Status conflict refers to disagreements about individuals’ relative positions in a group’s social hierarchy (Bendersky & Hays, 2012). Since status is a finite social and relative resource, conflicts over it often leads to zero-sum outcomes; one person’s gain in status results in another’s loss (Gould, 2003; Homans, 1961). Thus, this study focuses on status conflict within groups as a vital structural factor that affects employee voice. Status can only be established if all group members agree on the hierarchy. Therefore, if there is a disparity in perceived status conflict among members, it indicates that uncertainty or a lack of legitimacy regarding status exists within the group. As such, when an employee perceives more or less status conflict than their peers, the discrepancy is typically externally attributed to the social environment (“this hierarchy is unfair” or “our team dynamics are unstable”). Such external attributions evoke outwardly focused emotions such as anger or moral conviction, which motivate efforts to restore equity or assert clarity about the status order. From this perspective, attitudinal dissimilarity in status conflict may spur employees to voice their opinions in order to defend, challenge, or redefine the group’s status structure. In our framework, dissimilarity in perceived status conflict is measured at the individual level, but it concerns a structural property of the group—the status hierarchy. Thus, it represents an individual’s attitudinal deviation regarding how much status conflict exists within the team. For all three focal constructs—psychological ownership, job stress, and status conflict—we measure dissimilarity as an objectively calculated deviation from the scores of other team members. The underlying items capture employees’ subjective attitudes and perceptions (e.g., perceived ownership or perceived status conflict), but the dissimilarity indices themselves are computed using a standard dissimilarity formula. Therefore, when we refer to “dissimilarity in perceived status conflict,” we denote an objective measure of how much an employee’s perception of status conflict deviates from those of their teammates, rather than a subjective sense of felt dissimilarity. Thus, dissimilarity in perceived status conflict, calculated as the deviation of their perceived status conflict from that of their team members, will derive voice behavior.

**H3.**  *An employee’s dissimilarity in perceived status conflict will be positively related to their voice behavior.*

## 3. Method

### 3.1. Sample and Procedures

The current study’s data were collected from team members and team leaders from organizations in South Korea. Before allocating the survey, we received an organizational chart and team roster. We clarified to the participants and HR managers that employees could voluntarily participate in the study and assured them of the anonymity of their responses. To match team members’ self-reports with team leaders’ ratings while preserving anonymity, the HR department assigned a unique numerical code to each employee. This code was printed on both the employee questionnaire and the corresponding leader questionnaire, without including any names or personally identifying information. Only the research team had access to the anonymous codes, and HR did not see the completed surveys. This procedure allowed us to merge employee and leader data at the individual level without compromising confidentiality. This study collected data related to employees’ demographic characteristics and attitudes. Of the 306 individuals, 202 team members responded to the survey questionnaires (80.7% response rate). We also requested team leaders to evaluate their employees’ voice behavior to avoid common method bias (Podsakoff et al., 2003). Given the importance of precise results, this study removed unreliable responses as excessive missing data and outliers.

The final sample included 202 employees across 39 work groups, 69.6% of whom were male and 30.4% female, and an average team tenure of 18.8 months (SD = 47.6 months). The age distribution was categorized into five groups: 19.6% of participants were in their 20 s, 33.3% were in their 30 s, 20.1% were in their 40 s, 10.8% were in their 50 s, and 16.2% were aged 60 and above. The average team size for the analyses was 5.23. The original English items were translated into Korean using a standard back-translation procedure. Three bilingual researchers independently translated the survey into Korean and back-translated it into English. Discrepancies were discussed and resolved to ensure semantic equivalence. The final Korean version of the questionnaire was used for data collection with both employees and team leaders.

### 3.2. Measures

Employee Voice. Team leaders assessed the promotive and prohibitive voice behaviors of their supervised team members using the scale developed by Liang et al. (2012). The original scale distinguishes between promotive voice (e.g., “This employee proactively suggests new projects that are beneficial to the work unit”) and prohibitive voice (e.g., “This employee speaks up honestly when they see things that might negatively affect the unit”). All items were rated on a 5-point Likert scale (1 = never, 5 = very frequently). Although the original measure distinguishes promotive and prohibitive voice, we followed prior research that has treated voice as a higher-order, unified construct representing employees’ general tendency to speak up (e.g., Chamberlin et al., 2017; Maynes & Podsakoff, 2014). Consistent with this approach, we conducted confirmatory factor analyses comparing a one-factor model and a two-factor model. Results indicated that the one-factor model clearly exhibited better comparative fit with improvements in CFI (ΔCFI = 0.020), supporting the use of an aggregated voice construct. We therefore used the averaged score across all items as our primary indicator of employee voice.

Dissimilarity in Psychological Ownership. This study measured three items used in the sense of belongingness criteria of psychological ownership (e.g., “I feel a very high degree of personal ownership for this organization”) using a 5-point scale: (from 1 = strongly disagree, to 5 = strongly agree) (Avey et al., 2009). The dissimilarity in psychological ownership score is the difference between an individual and all other employees in the work team, using a formula similar to that used by Tsui et al. (1992). It is the square root of the summed squared differences between an individual $S_{i}$’s value on a psychological ownership variable and the value on the same variable for every other individual $S_{j}$ in the sample for the work unit, divided by the total number of respondents in the unit (*n*). The following formula was used for the dissimilarity calculation:

1n∑j=1n(Si−Sj)212

Dissimilarity in Job stress. Job stress is defined as an individual’s psychological response to aspects of the work environment that appear emotionally and physically threatening (Parker & DeCotiis, 1983). In this study, we measured the job-related anxiety dimension of the original Parker and DeCotiis (1983) 13-item scale, which consists of five items. We selected four items from this subdimension because one item exhibited substantially lower factor loading in our preliminary analysis. The four retained items captured core facets of job-related strain (e.g., “I feel a great deal of stress because of my job”) and demonstrated strong loadings on a single factor (all λs > 0.62). The internal consistency of the shortened four-item scale was acceptable (α = 0.78). All items were rated on a 5-point Likert scale (1 = strongly disagree, 5 = strongly agree). The dissimilarity in job stress scores was calculated using the standard dissimilarity formula described above.

Dissimilarity in Status conflict. The measures for status conflict assessed individuals’ perceptions of disputes regarding relative status positions within their group’s social hierarchy. Consistent with prior research on status conflict (Bendersky & Hays, 2012), we asked participants to respond to items assessing disputes regarding relative status positions within their group’s social hierarchy (e.g., “My team members disagreed about the relative value of members’ contributions”) using a 5-point scale (1 = strongly disagree, 5 = strongly agree). The dissimilarity in perceived status conflict scores was also evaluated using the dissimilarity formula.

Control variables. We statistically controlled for several variables relevant to this research model. We first controlled for gender (female = 0, male = 1), as males may have shown a greater willingness to engage in individual speaking up with a higher sense of self-efficacy (Morrison, 2023). In addition, we controlled for age and team tenure, because the employee could have a responsibility that engaged them in voice behavior (Stamper & Van Dyne, 2001). This study also controlled for psychological safety, which is positively associated with employee voice (Liang et al., 2012). Psychological safety is the extent to which individuals feel secure in expressing themselves openly at work. Members feel comfortable speaking up freely (Carmeli & Gittell, 2009). It was measured using Edmondson’s (1999) items (e.g., “it is safe to take a risk in this organization”) using a 5-point scale (from 1 = strongly disagree, to 5 = strongly agree). Because our sample was drawn from South Korean organizations and was ethnically homogeneous, we did not control for ethnicity. We note this as a scope condition of our findings.

## 4. Results

### 4.1. Descriptive Statistics

Table 1 presents the means, standard deviations, and correlations for all variables in the study. The correlation analysis revealed that the dissimilarity in status conflict was positively correlated with employee voice (*r* = 0.145, *p* < 0.05), while dissimilarity in psychological ownership (*r* = −0.086, ns) and job stress (*r* = −0.090, ns) did not show a significant relationship with employee voice. As to validity and reliability analyses, the measurement model showed acceptable fit: χ^2^ = 240.50, *p* < 0.01, CFI = 0.94, TLI = 0.93, RMSEA = 0.05. Cronbach’s α values were 0.77 (psychological safety), 0.70 (psychological ownership), 0.78 (job stress), 0.80 (status conflict), and 0.92 (voice).

### 4.2. Hypothesis Testing

We tested our hypotheses using hierarchical regression analysis using SPSS 20.0 for Windows. Table 2 presents the results of hierarchical regression analysis. Model 1 presents the analysis with control variables only, Model 2 includes independent variables: dissimilarity in psychological ownership, job stress, and status conflict. In the first step, we entered seven control variables. Results showed that gender (*b* = 0.352, *p* < 0.05), age (*b* = −0.256, *p* < 0.05), and psychological ownership (*b* = −0.345, *p*< 0.01) were significantly related to employee voice (Model 1 in Table 2).

Model 2 in Table 2 reveals the result of the hypotheses. H1 predicts that the dissimilarity in psychological ownership decreases employee voice, we found that dissimilarity in psychological ownership has a significantly negative effect on employee voice (*b* = −0.238, *p* < 0.0). Thus, H1 was supported. H2 predicts that dissimilarity in job stress is negatively associated with employee voice behavior. As shown in Model 2 in Table 2, we found that the coefficient of dissimilarity in job stress was negative (*b* = −0.122, *p* < 0.01), supporting H2. Hypothesis 3 predicts that dissimilarity in status conflict is positively associated with employee voice. Model 2 in Table 2 indicates that dissimilarity in status conflict increases employee voice (*b* = 0.200, *p* < 0.05). Therefore, H3 was also supported.

## 5. Discussion

This study sought to understand how attitudinal dissimilarity within work groups shapes employee voice. Drawing on social comparison and attribution perspectives, we distinguished between individual-level dissimilarities (psychological ownership and job stress) and perceived status conflict. Consistent with our predictions, dissimilarity in psychological ownership and job stress reduced voice, whereas dissimilarity in perceived status conflict increased voice. Below, we situate these findings in relation to prior research on voice, silence, and diversity, highlighting how our results extend the existing literature. First, our findings resonate with and extend prior research showing that employees who perceive themselves as different from their peers often anticipate higher interpersonal risk, which inhibits speaking up (Morrison, 2011; Bowen & Blackmon, 2003). The negative effects of attitudinal dissimilarity in psychological ownership and job stress align with the broader silence literature documenting that individuals who feel misaligned with group norms tend to withdraw or self-censor (Dehkharghani et al., 2023). By showing that these forms of deep-level misalignment predict lower voice, our study offers empirical support for the argument that minority positions can lead to behavioral inhibition when differences are interpreted as personal shortcomings rather than situational features. At the same time, our results extend research suggesting that minority positions can also motivate voice when differences are attributed externally or to system-level issues. Prior studies have shown that disagreements over hierarchy, legitimacy, or relational standing can spur individuals to challenge the group’s status quo (Bendersky & Hays, 2012; Imam et al., 2022). Our finding that dissimilarity in perceived status conflict increases voice adds nuance to this literature by demonstrating that it is not status conflict per se but perceived deviation in status conflict that activates outwardly oriented responses. This extends recent arguments that conflict around status and hierarchy can sometimes provoke constructive challenge and learning (Bendersky & Hays, 2017; Kang & Lee, 2021). Second, our results contribute to the diversity literature by providing more granular evidence on when deep-level dissimilarity suppresses versus stimulates employee expression. Whereas much of the deep-level diversity research has highlighted its effects on affective and relational outcomes (Triana et al., 2021b; Şahin et al., 2019), fewer studies have linked attitudinal misalignment to behavioral consequences such as voice. Our study shows that attitudinal differences are not uniformly harmful; instead, the interpretive meaning attached to the difference—internal versus external attribution—drives whether dissimilarity leads to silence-oriented withdrawal or voice-oriented expression. In doing so, we advance recent calls to unpack the cognitive and attributional processes underlying diversity effects in work groups (Qu et al., 2024). Third, our findings also speak to the distinction between promotive and prohibitive forms of voice. Although our study used an aggregate measure, the observed patterns are consistent with recent evidence that perceived interpersonal risk suppresses both forms of voice, whereas structural misalignment can trigger more critical or change-oriented suggestions (Liang et al., 2019). Future research should examine whether dissimilarity in status conflict is especially likely to foster prohibitive voice aimed at correcting problematic dynamics in the group. Finally, our findings hold important implications for understanding how employees navigate attitudinal minority positions. Prior work describes minority status as a constraint due to reduced belonging and increased uncertainty. Our study suggests that attitudinal minorities are not universally silent; rather, they may become “boisterous minorities” when their differences signal structural issues rather than personal deficiencies. This distinction underscores the need for future multilevel research on when group disagreement fosters learning rather than avoidance. Together, these comparisons position our work more clearly within current debates on voice, silence, and diversity. By showing that attitudinal dissimilarity can generate both withdrawal and expression depending on the level and meaning of the difference, our study refines theoretical assumptions about the role of minority positions in shaping proactive behavior at work.

### 5.1. Theoretical Contributions

Taken together, our findings offer several important theoretical contributions to the literature on employee voice and workplace diversity. First, this study expands the employee voice literature by moving beyond motivational antecedents such as psychological safety, leadership, and individual proactivity. We show that employees’ decisions to speak up or stay quiet are also shaped by relational positioning—how they perceive their attitudinal alignment or misalignment with others in the group. Voice is therefore not only a matter of “can” or “will,” but also of where one stands in relation to others. Second, by integrating social comparison and attribution theories, our study illuminates the cognitive processes through which employees interpret their differences. We demonstrate that the direction of attribution—whether internal or external—functions as a psychological switch between attenuating their willingness to voice and speaking up. This theoretical integration links two previously distinct perspectives, revealing how social comparison triggers attributional reasoning that guides behavior. Third, our findings reframe dissimilarity as a double-edged social cue rather than a uniformly negative feature. Dissimilarity in individual attitudes fosters internalized self-focus and attenuated their willingness to voice, whereas dissimilarity in structural perceptions stimulates outward-oriented voice. In doing so, we move the diversity literature toward a more nuanced understanding of how differences can both constrain and activate constructive expression. Fourth, this research introduces the conceptual distinction between invisible minorities and boisterous minorities as two possible responses to attitudinal minority positions. Invisible minorities are employees whose attitudinal dissimilarity leads them to interpret their difference through self-focused attributions (e.g., “something is wrong with me”), heightening self-consciousness and making them less likely to speak up. Boisterous minorities are employees whose attitudinal dissimilarity—particularly in perceptions of status conflict—leads them to interpret their difference as reflecting systemic issues (e.g., “this hierarchy is unfair”), thereby motivating them to voice concerns in order to defend or challenge the status quo. We do not empirically classify individuals into these two categories; rather, we use these conceptual labels to describe the contrasting behavioral patterns implied by our findings: individual-level dissimilarities in psychological ownership and job stress are associated with lower voice, whereas dissimilarity in perceived status conflict is associated with higher voice. This duality enriches the understanding of how individuals navigate difference within social comparison processes and offers a new vocabulary for describing behavioral diversity in groups. Fifth, our study contributes to deep-level diversity research by empirically linking attitudinal dissimilarity to behavioral outcomes. While demographic diversity has long been the focus, our results underscore that difference in how employees think and feel can be even more consequential for voice. This advances the field by empirically connecting deep-level diversity to one of the most critical proactive behaviors in organizations.

### 5.2. Practical Implications

Our findings can be translated into concrete guidance for managers. First, managers should recognize that silence is not necessarily a sign of disengagement but may reflect a sense of internalized difference. Detecting such “invisible minorities” requires paying attention to subtle cues—employees who consistently withdraw, self-censor, or hesitate to contribute may be struggling with perceived misalignment rather than lack of motivation. Importantly, our dissimilarity measure captures absolute deviation rather than direction: employees who feel either much more or much less ownership than others may both withdraw from speaking up. This suggests that what matters for voice is not whether an employee is “high” or “low” on ownership in absolute terms, but whether they perceive themselves as standing apart from the team norm and internally attribute this difference to personal shortcomings. Managers should therefore pay attention to employees whose engagement patterns diverge—either very invested or notably detached—and proactively invite their input in ways that reduce self-focused anxiety about being different. Second, managers should cultivate climates that normalize difference. Our results complement recent evidence that leadership practices and team climates can shape whether diverse voices are heard or marginalized (Weiss et al., 2018; Yin & Liu, 2022). When variations in attitudes, stress, or perceptions are accepted as natural, employees are less likely to attribute their differences to personal shortcomings. This normalization can reduce self-blame and foster open, psychologically safe dialogue. Third, leaders should learn to manage status conflict constructively. Recent work further suggests that status-related tensions need not always be detrimental and may sometimes stimulate constructive challenge and creativity when managed appropriately (Bendersky & Hays, 2017; Imam et al., 2022). Our finding that dissimilarity in perceived status conflict is positively related to voice aligns with this more nuanced view. Because dissimilarity in perceived status conflict can motivate voice, managers can channel this energy toward collective learning rather than rivalry. Structured discussions and transparent criteria for recognition can convert status tension into a healthy source of debate and improvement. Fourth, supervisors can support attitudinal minorities by framing their input as valuable for group learning and emphasizing affective trust. When employees believe they can speak up without jeopardizing relationships, they are more likely to transform their unique perspectives into constructive contributions. Fifth, human resource systems should incorporate measures of deep-level attitudes—such as psychological ownership, job stress, or perceptions of hierarchy—into surveys and team assessments. Tracking these attitudinal indicators alongside demographic data enables organizations to identify relational mismatches that might otherwise remain invisible.

As such, these findings echo recent work showing that inclusive climates and leader behaviors can amplify employees’ willingness to speak up despite differences (Weiss et al., 2018; Yin & Liu, 2022). Managers should therefore not aim to reduce attitudinal diversity but rather create norms that legitimize differences as valuable sources of insight.

### 5.3. Limitations and Future Research

As with any field study, this research has limitations that offer avenues for future exploration. First, the cross-sectional design restricts causal inference. Future studies could employ longitudinal or experimental designs to capture dynamic shifts in attribution and voice over time. Second, our sample was drawn from South Korean organizations; cultural norms emphasizing harmony may amplify the tendency toward silence among attitudinal minorities. Replications in more individualistic or egalitarian contexts would strengthen generalizability. Third, we focused on cognitive attributions but did not measure the specific emotional mediators (e.g., guilt, anger, moral conviction) that likely link attribution to behavior. Examining these emotional pathways would deepen understanding of the underlying mechanisms. Fourth, we examined voice at the individual level, yet dissimilarity may also shape collective voice through contagion or modeling processes—a promising direction for multilevel research. Finally, although our primary analyses focused on an overall measure of voice, the Liang et al. (2012) scale distinguishes between promotive and prohibitive voice. Future research could explore boundary conditions that differentially shape how attitudinal dissimilarity influences these two types of voice. Identifying such moderators would provide a more nuanced understanding of when and why different forms of “speaking up” are affected by one’s relative attitudinal position within the group. Furthermore, future research could meaningfully extend this work by explicitly considering the direction of dissimilarity (e.g., being higher or lower than others in the team). Whereas our study focuses on absolute deviation from the team mean, employees who score substantially higher or lower than their teammates could experience different attributional processes and psychological reactions.

## 6. Conclusions

In a world where organizations increasingly value openness and inclusion, understanding when difference leads to silence versus expression is vital. This study demonstrates that employees’ reactions to their dissimilarity depend on how they attribute its cause—whether they look inward or outward, whether they self-protect or seek to correct the system. By revealing that attitudinal minorities can be either invisible or boisterous, our findings highlight the socially constructed nature of voice behavior. Ultimately, encouraging voice requires not only empowering individuals but also reshaping the relational and cognitive contexts that determine how difference is experienced and expressed within work groups.

## Figures and Tables

**Table 1:** Means, Standard Deviations, and Correlations ^a^.

Variable	Mean	s.d.	1	2	3	4	5	6	7	8	9	10
1. Gender	0.70	0.46										
2. Age	3.12	0.80	0.283 ***									
3. Team tenure	18.81	47.55	−0.055	0.280 ***								
4. Psychological ownership	3.57	0.75	0.065	0.189 *	0.013							
5. Job stress	3.03	0.85	0.095	−0.095	−0.054	−0.455 **						
6. Status conflict	2.04	0.68	0.065	−0.099	0.082	−0.136	0.174 *					
7. Psychological safety	3.72	0.63	−0.011	−0.009	−0.042	0.453 **	−0.137	−0.442 **				
8. Dissimilarity in psychological ownership	1.02	1.05	0.125	0.039	−0.101	−0.035	0.092	−0.008	0.071			
9. Dissimilarity in job stress	1.46	1.29	0.080	−0.123	−0.030	0.119	0.143 *	−0.006	−0.003	0.081		
10. Dissimilarity in status conflict	0.74	0.74	0.142 *	−0.144 *	−0.138	−0.101	0.013	0.244 **	−0.064	0.284 **	0.087	
11. Employee voice	3.53	0.65	0.171 *	−0.140	−0.026	−0.143	0.120	−0.008	0.132	−0.086	−0.090	145 *

^a^ n = 202, * *p* < 0.05, ** *p*< 0.01, *** *p* < 0.001 (two-tailed).

**Table 2:** Results of Regression Analyses Predicting Employee Voice ^a^.

Variable	Model 1	Model 2
Gender	0.352 * (0.153)	0.312 * (0.145)
Age	−0.256 * (121)	−0.213 (0.110)
Team tenure	0.007 (0.004)	0.005 (0.003)
Psychological ownership	−0.345 ** (128)	−0.204 (0.121)
Job stress	−0.073 (0.094)	0.008 (0.088)
Status conflict	−0.011 (0.121)	−0.037 (0.110)
Psychological safety	0.222 (0.143)	0.216 (0.129)
Dissimilarity in psychological ownership		−0.238 ** (0.081)
Dissimilarity in job stress		−0.122 ** (0.046)
Dissimilarity in status conflict		0.200 * (0.083)
R^2^	0.438	0.373
Adjusted R^2^	0.122	0.292
F	2.749	4.637
∆R^2^	0.192	0.181
∆F	2.759	7.500

^a^ n = 202. Values represent unstandardized coefficients; Standard errors are noted in parentheses. * *p* < 0.05, ** *p* < 0.01 (two-tailed).

## Data Availability

The data of this study are available from the corresponding author upon reasonable request.
